# Overexpression of Modified *CENH3* in Maize Stock6-Derived Inducer Lines Can Effectively Improve Maternal Haploid Induction Rates

**DOI:** 10.3389/fpls.2022.892055

**Published:** 2022-04-11

**Authors:** Dexuan Meng, Haishan Luo, Zhaobin Dong, Wei Huang, Fang Liu, Fenghai Li, Shaojiang Chen, Haiqiu Yu, Weiwei Jin

**Affiliations:** ^1^College of Agronomy, Shenyang Agricultural University, Shenyang, China; ^2^State Key Laboratory of Plant Physiology and Biochemistry, Key Laboratory of Crop Heterosis and Utilization, National Maize Improvement Center, Ministry of Education, College of Agronomy and Biotechnology, China Agricultural University, Beijing, China; ^3^Department of Agronomy, College of Agriculture and Resources and Environmental Sciences, Tianjin Agricultural University, Tianjin, China

**Keywords:** maize, *CENH3*, Stock6, haploid inducer line, haploid induction rate

## Abstract

Maize (*Zea mays*) doubled haploid (DH) breeding is a technology that can efficiently generate inbred lines with homozygous genetic backgrounds. Haploids are usually produced through *in vivo* induction by haploid inducer lines in maize. Currently, two approaches are usually used to develop maize haploid inducer lines. One is through the conventional breeding improvement based on the Stock6 germplasm, and this strategy is extensively used to induce maternal haploids in commercial maize DH breeding. Another strategy, newly developed but less utilized so far, is by genetic manipulation of the *Centromeric Histone3 (CENH3)* in regular lines. However, whether both approaches can be combined to develop the haploid inducer line with higher maternal haploid induction rate (HIR) has not been reported. In this study, we manipulated the Stock6-derived inducer lines by overexpressing maize CENH3 fused with different fluorescent protein tags and found that the engineered Stock6-derived lines showed an obvious increase in the maternal HIR. Intriguingly, this above strategy could be further improved by substituting a tail-altered *CENH3* for the full-length *CENH3* in the tagged expression cassette, resulting in a maternal HIR up to 16.3% that was increased by ~6.1% than Stock6-derived lines control. These results suggested that integration of two *in vivo* haploid induction methods could rapidly and effectively improve the maternal HIRs of maize Stock6-derived inducer lines, and provided a potentially feasible solution for further optimizing the process of commercial maize DH breeding.

## Introduction

Maize DH breeding technology allows breeders to develop homozygous lines within one generation (Chang and Coe, 2009). In contrast to conventional breeding, which requires six to eight generations to develop an inbred line *via* extensive selfing or backcrossing, DH breeding can effectively reduce time and resource consumption and thus is gradually becoming important in modern maize breeding (Geiger and Gordillo, 2009; Chaikam et al., 2019).

Haploids can be induced by several *in vitro* or *in vivo* methods, and *in vivo* maternal haploid induction (HI) based on Stock6-derived haploid inducer lines is a commonly used approach in commercial maize breeding (Chaikam et al., 2019; Kalinowska et al., 2019). In 1959, the ancestral haploid inducer line Stock6, which exhibits only a 2.3–3.2% maternal HI rate (HIR), was first reported (Coe, 1959). In the following decades, Stock6 has been constantly improved and used to breed many derivative lines with various genetic backgrounds (Chalyk, 1999; Chen and Song, 2003; Röber et al., 2005; Chang and Coe, 2009; Rotarenco et al., 2010). These Stock6-derived lines have better agronomic traits and higher maternal HIRs (7–16%) and therefore have been widely applied in commercial maize breeding systems. Currently, eight quantitative trait loci (QTLs) have been identified to be responsible for the maternal HI ability of maize haploid inducer lines. *quantitative haploid induction rate 1 (qhir1)* is one of the major QTLs, explaining ~66% of the genetic variance, and is essential for the HI capacity of Stock6 and its derivative lines (Prigge et al., 2012; Dong et al., 2013). In 2017, the causal gene in the *qhir1* locus in maize was isolated and named *Phosphlipase A1* [*ZmPLA1*, also known as *matrilineal (MATL)* and *not like dad (NLD)*; Gilles et al., 2017; Kelliher et al., 2017; Liu et al., 2017]. In 2019, researchers isolated another pollen-specific HI gene, *ZmDMP*, which is located in the second major QTL, *quantitative haploid induction rate 8 (qhir8*; Zhong et al., 2019). Interestingly, the causative allele of *ZmDMP* did not originate from Stock6 and is widely present in various germplasms. Moreover, knockout of *ZmDMP* in maize not only triggered HI but also significantly enhanced the HI capacity of inducer plant in the presence of *mtl*/*zmpla1*/*nld* mutation.

*CENH3*-mediated *in vivo* HI is another promising approach used for producing haploids in commercial breeding programs. *CENH3* encodes a centromere-specific variant of histone3 (H3), which is responsible for kinetochore formation and spindle attachment during mitosis and meiosis (Jiang et al., 2003; Burrack and Berman, 2012; Fukagawa and Earnshaw, 2014; McKinley and Cheeseman, 2016). The CENH3 protein contains two domains, namely, a highly variable N-terminal tail domain and a relatively conserved C-terminal histone fold domain (HFD; Maheshwari et al., 2015; McKinley and Cheeseman, 2016). In 2010, Ravi and Chan first found that expressing a series of green fluorescent protein-tagged CENH3 variants in Arabidopsis *cenh3* null mutants could not only rescue the embryo-lethal phenotype but also create a new HI ability in transgenic plants (Ravi and Chan, 2010). In particular, in the transgenic lines expressing CENH3 with the N-terminal tail altered, the HIR reached approximately 45% upon outcrossing with the wild-type plants as a female parent. In subsequent work, using TILLING and CRISPR/Cas9 technologies, researchers further determined that modification of the HFD of CENH3 could also create haploid inducer lines (Karimi-Ashtiyani et al., 2015; Kuppu et al., 2015, 2020). Similarly, the strategy of modifying the CENH3 protein has been transferred to maize and wheat and has been proved to successfully induce haploids, although the current HIR in other species is far lower than that in Arabidopsis (Kelliher et al., 2016; Kalinowska et al., 2019; Lv et al., 2020; Wang et al., 2021).

In maize DH breeding programs, improvement of the HIR remains one of the major objectives as it can further reduce breeding costs and improve breeding efficiency (Chaikam et al., 2019). Currently, although multiple HI ability-related loci have been identified, the genetic contribution of these loci seem to be limited, and it is difficult to further improve the HIRs of haploid inducer lines only using the conventional breeding strategy. To breed new haploid inducers with higher HIRs suitable for modern maize DH breeding, a new strategy, such as integration with genetic engineering technology, is needed to be developed. Here, we overexpressed the original or tail-altered CENH3 fused with different fluorescent proteins in Stock6-derived lines and bred a range of new haploid inducer lines by means of genetic engineering and molecular marker-assisted selection methods. Subsequent examination revealed that the maternal HIR of all Stock6-derived inducer lines overexpressing exogenous modified *CENH3* was significantly increased compared to that of the control lines. These results demonstrated that the two existing approaches of *in vivo* HI could be well integrated to effectively and rapidly improve the maternal HIRs of maize haploid inducer lines.

## Results

### Overexpression of Modified Maize *CENH3* Can Increase the Maternal HIRs of Stock6-Derived Inducer Lines

**Figure 1:**
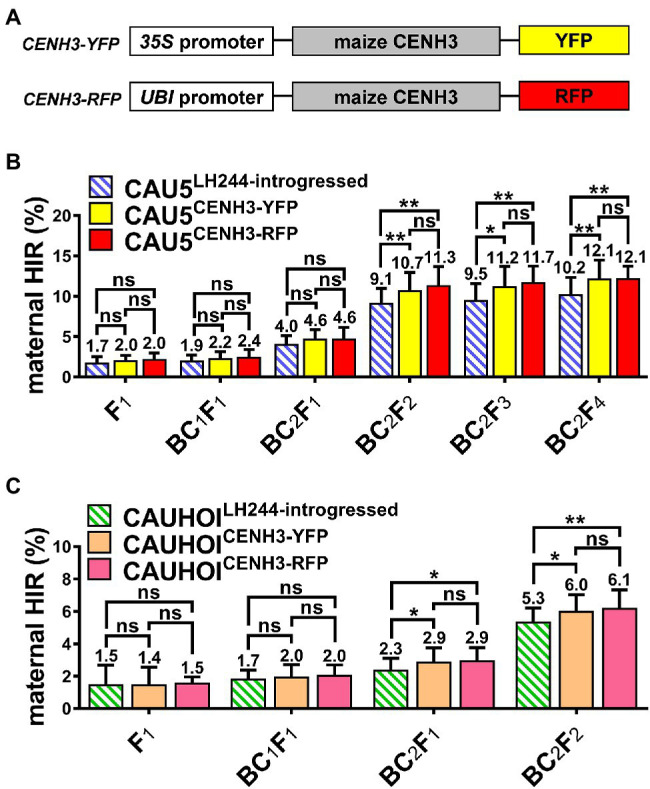
Overexpression of the fluorescent protein-tagged CENH3 in Stock6-derived inducer lines could increase the maternal HIR significantly. **(A)** Schematic diagram of *CENH3-YFP* and *CENH3-RFP* overexpression vectors. **(B)** Statistical analysis for maternal HIR of the CAU5-backcrossed inducer lines CAU5^LH244-introgressed^, CAU5^CENH3-YFP^, and CAU5^CENH3-RFP^ in consecutive breeding generation. **(C)** Statistical analysis for maternal HIR of the CAUHOI-backcrossed inducer lines CAUHOI^LH244-introgressed^, CAUHOI^CENH3-YFP^, and CAUHOI^CENH3-RFP^ in consecutive breeding generation. The number above the column in **(B,C)** indicates the average maternal HIR. Error bars indicate ± standard deviation (SD). Significant differences were analyzed by two-tailed Student’s *t*-tests (ns, not significant; ^*^p < 0.05; ^**^p < 0.01).

A previous study revealed that the expression of an exogenous modified *CENH3* in the native *cenh3* mutant plants could create haploid inducer lines with up to ~3.6% maternal HIR (Kelliher et al., 2016). Maize Stock6-derived haploid inducer lines are usually used as male parents to induce maternal haploids (Kalinowska et al., 2019). To determine whether overexpression of an exogenous *CENH3* gene (*Zm00001d038533*) fused with a bulky tag in the Stock6-derived lines would have a positive effect on their maternal HIRs, we first generated a construct driven by the *CaMV 35S* promoter fusing maize native CENH3 with a yellow fluorescent protein (YFP; termed *CENH3-YFP*) and introduced it into the transformable line LH244 (termed LH244^CENH3-YFP^; Figure 1A). However, no haploid was detected in the reciprocal cross between LH244^CENH3-YFP^ and tester line (the hybrid Zhengdan958), which indicated that overexpression of YFP-tagged CENH3 in the regular line did not produce a maternal or paternal HI ability (Table 1; Supplementary Figure 1).

**Table 1:** Maternal HIR test of the modified-*CENH3* overexpression lines.

Cross combination		Total seeds	Haploids	HIR (%)
Zhengdan958 (♀) ⨯	LH244^CENH3-YFP^	845	0	0
	LH244^CENH3-RFP^	674	0	0
	HiII^C-tailswap-YFP^	1,034	0	0
	LH244^M-tailswap-RFP^	1,203	0	0
LH244^CENH3-YFP^	Zhengdan958 (♂)	885	0	0
LH244^CENH3-RFP^ ⨯		823	0	0
HiII^C-tailswap-YFP^		1,131	0	0
LH244^M-tailswap-RFP^		944	0	0

Subsequently, this LH244^CENH3-YFP^ transgenic line was backcrossed with the Stock6-derived inducer line CAU5 (a maternal haploid inducer line, developed by China Agricultural University; Xu et al., 2013) twice and then continuously selfed to breed a new inducer line (termed CAU5^CENH3-YFP^; Figure 1B). Since the *qhir1* locus is essential for the HI ability of Stock6-derived germplasms, we simultaneously screened the *qhir1* locus using molecular markers during the selection process of the CAU5^CENH3-YFP^ inducer lines. In each generation, 25 plants containing both the *qhir1* locus and *CENH3-YFP* vector were randomly screened for testing the maternal HIR with three replications (each inducer plant was used to pollinate three tester plants of Zhengdan958). In the next generation, the seeds from three plants with the highest maternal HIRs were selected for planting. Moreover, the transformable line LH244 was also backcrossed with CAU5 as the negative controls (termed CAU5^LH244-introgressed^) using the same selection strategy (Figure 1B).

According to the statistical data (Figure 1B; Supplementary Table 1), after a slight improvement in the HIR of the CAU5^CENH3-YFP^ line during early generations (from F_1_ to BC_2_F_1_ generations), and was significantly increased from the BC_2_F_2_ generation, and reached ~12.1% in BC_2_F_4_, which was 1.9% higher than that of the control line CAU5^LH244-introgressed^ (Figure 1B). Since the progenies after the BC_2_F_1_ generation (after three backcrosses) theoretically contained at least 87.5% of the genetic background of the recurrent parent (CAU5), the results of the HIR test indicated that overexpression of a CENH3-YFP chimeric protein in CAU5 might enhance its maternal HI capacity. To verify this, the LH244^CENH3-YFP^ transgenic line was backcrossed with another Stock6-derived line, CAUHOI (a maternal haploid inducer line, developed by China Agricultural University, with high kernel oil content; Li et al., 2009), creating the CAUHOI^CENH3-YFP^ inducer line (Figure 1C). After three backcross generations and one selfing generation, we also observed a higher HIR as illustrated by using CAU5 as the backcross donor (Figure 1C; Supplementary Table 2). In addition, we also introduced the YFP empty vector into the CAU5 inducer line (termed CAU5^35S::YFP^) and revealed that overexpressing YFP alone could not improve the maternal HIR (Supplementary Figure 2; Supplementary Table 1). In summary, these results indicate that overexpression of YFP-tagged CENH3 in the Stock6-derived inducer lines could increase their HIRs.

### Different Tagged-*CENH3* or Different Transformable Lines Show Parallel Increase in the Maternal HIR

To investigate whether different fluorescent protein tags in the CENH3 fusion protein would affect the maternal HIR, we generated another modified *CENH3* overexpression vector with a red fluorescent protein tag (termed *CENH3-RFP)* and introduced it into CAU5 and CAUHOI inducer lines (termed CAU5^CENH3-RFP^ and CAUHOI^CENH3-RFP^; Figure 1A). The results showed that the maternal HIRs of the two newly bred inducer lines containing the *CENH3-RFP* vector were also significantly increased relative to those of their control lines (CAU5^LH244-introgressed^ and CAUHOI^LH244-introgressed^; Figures 1B,C; Supplementary Tables 1, 2). However, the extent of the increase in *CENH3-RFP*-overexpressing lines was not obviously different compared to that in *CENH3-YFP*-overexpressing lines in every generation (Figures 1B,C; Supplementary Tables 1, 2).

**Figure 2:**
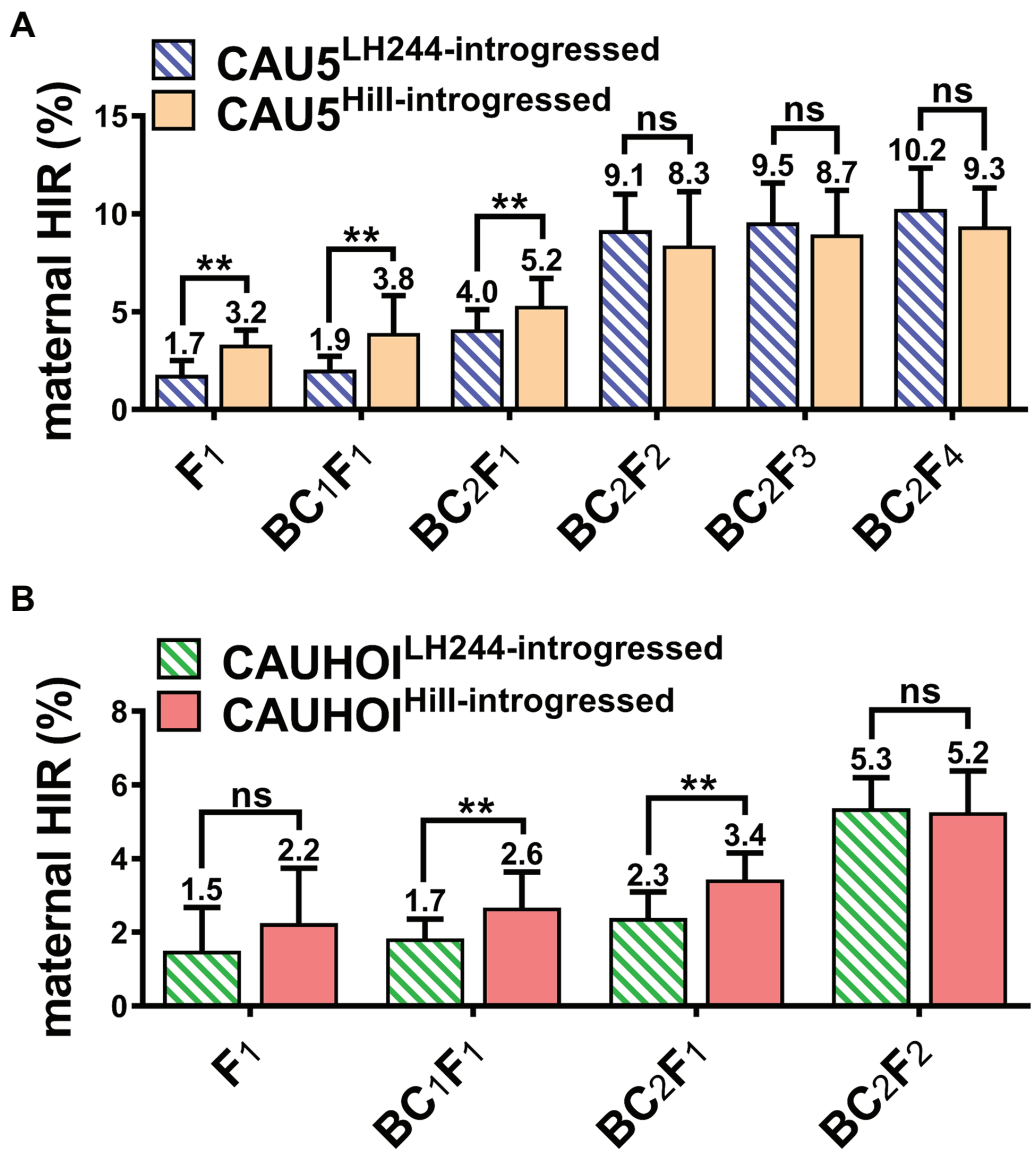
Little effect on the maternal HIR of the ultimately bred inducer lines using different genetic background transgenic receptors. **(A)** Statistical analysis for maternal HIR of the inducer lines CAU5^LH244-introgressed^ and CAU5^HiII-introgressed^ at each generation from F_1_ to BC_2_F_4_. **(B)** Statistical analysis for maternal HIR of the inducer lines CAUHOI^LH244-introgressed^ and CAUHOI^HiII-introgressed^ at each generation from F_1_ to BC_2_F_2_. The number above the column in **(A,B)** indicates the average maternal HIR. Error bars indicate ±SD. Significant differences were analyzed by two-tailed Student’s *t*-tests (ns, not significant; ^**^p < 0.01).

Furthermore, we used another transformable line, HiII, to study whether the genetic background of the transgenic receptor had an effect on the maternal HIR of the subsequently bred inducer. The transformable line HiII was backcrossed with the inducer lines CAU5 and CAUHOI according to the previous strategy. Interestingly, the HIRs of the inducer lines with a HiII genetic background (termed CAU5^HiII-introgressed^ and CAUHOI^HiII-introgressed^) were significantly higher than those of the lines with an LH244 genetic background at early breeding stages (from the F_1_ generation to the BC_2_F_1_ generation; Figures 2A,B; Supplementary Tables 1, 2). However, after self-pollination, the HIRs of the LH244 introgressed lines increased rapidly, leading to a slight difference in HIRs between the two genetic backgrounds in later breeding stages (after the BC_2_F_1_ generation; Figures 2A,B; Supplementary Tables 1, 2).

### Overexpression of Tail-Altered *CENH3* Could Further Increase the Maternal HIRs of Stock6-Derived Inducer Lines

**Figure 3:**
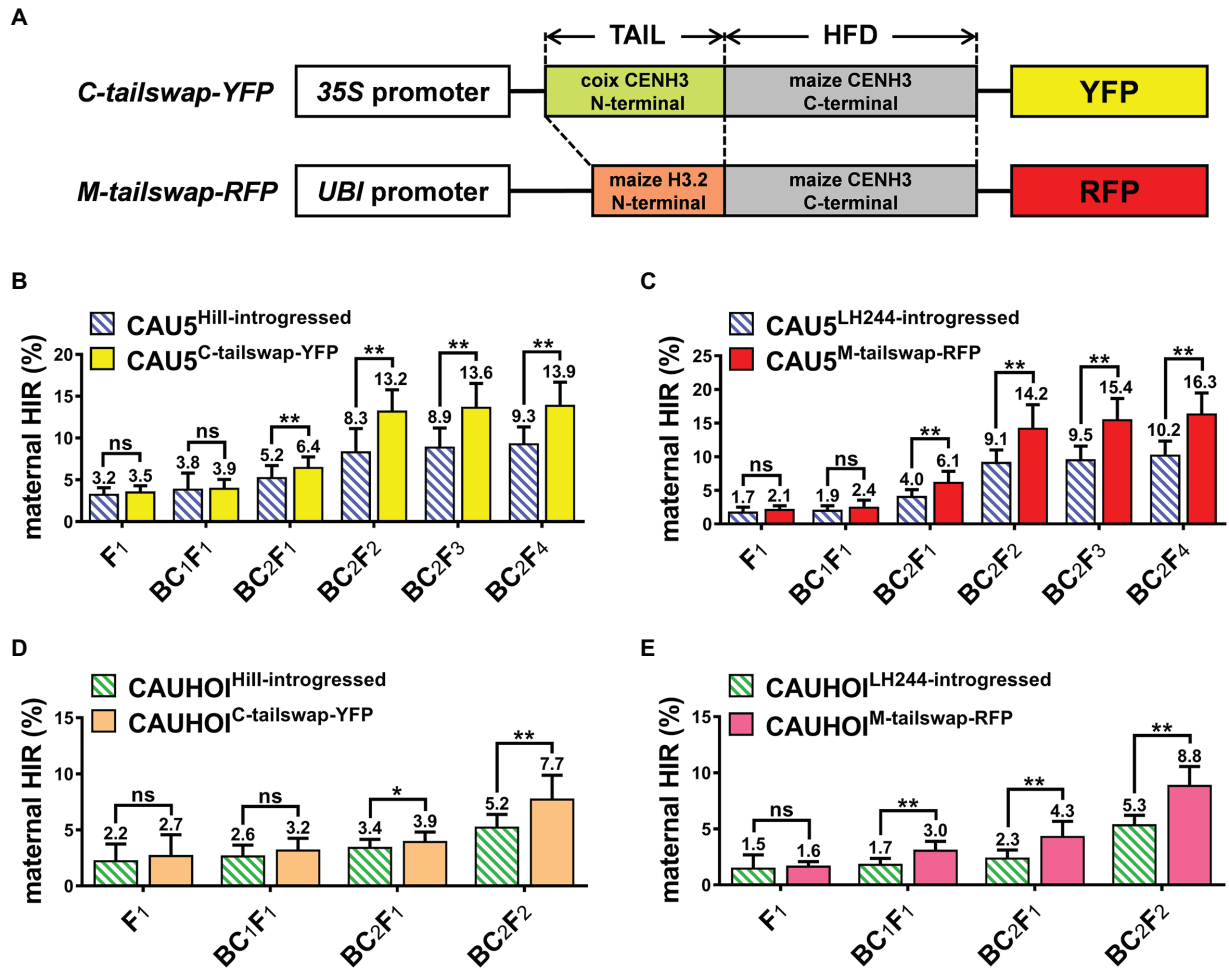
Modification of the N-terminal tail of the exogenous CENH3 chimeric protein could further increase the maternal HIRs of Stock6-derived inducer lines. **(A)** Schematic diagram of tail-altered *CENH3* overexpression vectors *C-tailswap-YFP* and *M-tailswap-RFP*. “TAIL” and “HFD” indicate the N-terminal tail and the C-terminal histone fold domain of CENH3. **(B)** Statistical analysis for maternal HIR of the CAU5-backcrossed inducer lines CAU5^HiII-introgressed^ and CAU5^C-tailswap-YFP^ in consecutive breeding generation. **(C)** Statistical analysis for maternal HIR of the CAU5-backcrossed inducer lines CAU5^LH244-introgressed^ and CAU5^M-tailswap-RFP^ in consecutive breeding generation. **(D)** Statistical analysis for maternal HIR of the CAUHOI-backcrossed inducer lines CAUHOI^HiII-introgressed^ and CAUHOI^C-tailswap-YFP^ in consecutive breeding generation. **(E)** Statistical analysis for maternal HIR of the CAUHOI-backcrossed inducer lines CAUHOI^LH244-introgressed^ and CAUHOI^M-tailswap-RFP^ in consecutive breeding generation. The number above the column in **(B–E)** indicates the average maternal HIR. Error bars indicate ±SD. Significant differences were analyzed by two-tailed Student’s *t*-tests (ns, not significant; ^*^p < 0.05; ^**^p < 0.01).

CENH3 contains a fast-evolving N-terminal tail domain that varies greatly even between closely related species and might be involved in speciation (Maheshwari et al., 2015). Previous studies have revealed that modification of the N-terminal sequence of exogenous CENH3 could further enhance the HI ability in transgenic plants compared to plants expressing exogenous CENH3 with the original sequence (Ravi and Chan, 2010; Kelliher et al., 2016). Our above results showed that overexpression of fluorescent protein-tagged CENH3 in Stock6-derived lines could significantly increase their HIRs. To explore whether changing the CENH3 protein sequence would further increase the maternal HIR, we modified the two above vectors (*CENH3-YFP* and *CENH3-RFP*) by replacing their N-terminal tail sequence with the N-terminal tail sequence of coix (*Coix lacryma-jobi*, a grass closely related to maize) CENH3 and the N-terminal tail sequence of maize HISTONE3.2 (H3.2; Figure 3A; Jiao et al., 2017; Liu et al., 2020). The two new constructs were named *C-tailswap-YFP* and *M-tailswap-RFP* and were transformed into the HiII and LH244 receptor lines, respectively (Figure 3; Supplementary Figure 1). Similar to the above original *CENH3* transgenic lines, neither HiII^C-tailswap-YFP^ nor LH244^M-tailswap-RFP^ lines showed any HI ability on their own (Table 1). Then, the same strategy was used to breed the new inducer lines containing both the *qhir1* locus and the target vector. Surprisingly, in the CAU5-backcrossed group, the HIRs of CAU5^C-tailswap-YFP^ and CAU5^M-tailswap-RFP^ lines showed a significant increase one generation earlier than that of the original *CENH3-*overexpressing group (CAU5^CENH3-YFP^ and CAU5^CENH3-RFP^; Figures 1B, 3B,C; Supplementary Table 1). Furthermore, the HIRs of both new inducer lines increased more rapidly from selfed generations. Notably, in the BC_2_F_4_ generation, the HIRs of CAU5^C-tailswap-YFP^ and CAU5^M-tailswap-RFP^ lines were ~ 4.6% and ~ 6.1% higher than those of their controls, reaching ~13.9% and ~ 16.3%, which were also ~1.8% and ~ 4.2% higher than those of CAU5^CENH3-YFP^ and CAU5^CENH3-RFP^, respectively (Figures 3B,C). Likewise, in the groups of CAUHOI-backcrossed inducer lines, the increasing trend of the HIR was similar to that in the CAU5-backcrossed lines (Figures 3D,E), and the HIRs of the ultimately bred lines (BC_2_F_2_ generation) were also significantly higher than those of the control lines and original *CENH3* overexpression lines (Figures 1C, 3D,E; Supplementary Table 2). Therefore, these results indicate that overexpression of the tail-altered CENH3 chimeric protein in the Stock6-derived lines background could further improve their maternal HI ability.

### Higher Maternal HIR Accompanied by Reduced Pollen Viability in Newly Bred Haploid Inducer Lines

**Figure 4:**
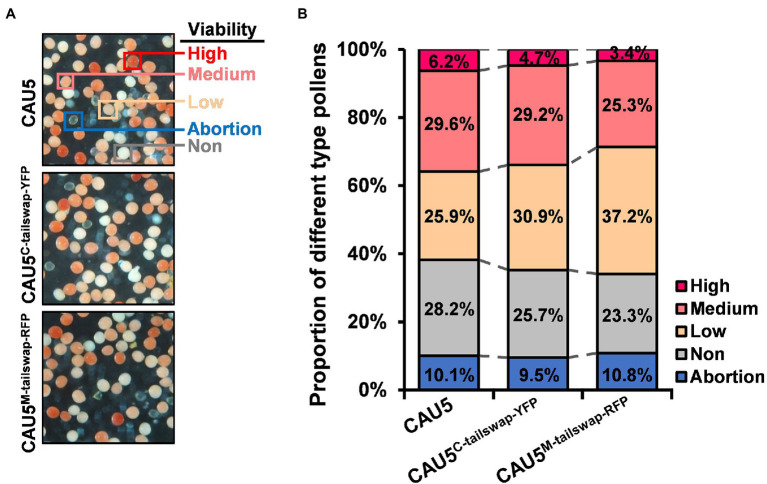
Analysis of pollen viability in the CAU5^C-tailswap-YFP^ and CAU5^M-tailswap-RFP^ inducer lines. **(A)** Pollen TTC staining of the inducer lines CAU5, CAU5^C-tailswap-YFP^, and CAU5^M-tailswap-RFP^. **(B)** Comparison of pollen viability among the CAU5, CAU5^C-tailswap-YFP^ and CAU5^M-tailswap-RFP^ inducer lines. More than 30,000 pollen grains were counted for each inducer line to analyze the proportion of different type pollens.

Previous studies revealed that the Stock6-derived inducer line has poor pollen competitive ability and contains a higher proportion of low-viability and non-viable pollen grains compared to the regular line (Xu et al., 2013; Li et al., 2017). In this study, we also detected pollen viability using the 2,3,5-triphenyltetrazolium chloride (TTC) staining method for two newly bred inducer lines with the highest maternal HIRs (CAU5^C-tailswap-YFP^ and CAU5^M-tailswap-RFP^; Figure 4A). Interestingly, the proportion of low-viability pollen in the two newly bred inducer lines was obviously increased, reaching ~30.9% and ~ 37.2%, compared with ~25.9% of CAU5 (Figure 4B), which was similar to the results of a previous study (Li et al., 2017). Since the variation trend of the proportion of low-viability pollen was well corresponding to the HIRs of three inducer lines, namely, CAU5 (~10.9%; Xu et al., 2013), CAU5^C-tailswap-YFP^ (~13.9%) and CAU5^M-tailswap-RFP^ (~16.3%; Figures 3B,C), it is speculated that the pollen viability of the inducer lines might be negatively correlated with their HIRs, possibly because pollen with HI ability usually has low viability.

## Discussion

In previous studies, the maternal HI ability of the maize Stock6-derived haploid inducer line was mainly controlled by eight QTLs, including two major and six minor QTLs (Prigge et al., 2012; Dong et al., 2013; Liu et al., 2015). In this study, since only one major QTL, *qhir1*, was screened for developing new inducer lines in both the experimental and control groups during the breeding process, the remaining QTLs related to HI ability may be heterozygous in the backcross generations; thus, the HIR was relatively low at the early breeding stage. However, the HIR increased markedly during the self-pollinated generations, which might be due to the homozygosity of these potential HI ability-related loci. Interestingly, although the HIRs of the newly bred inducer lines from the CAUHOI-backcrossed groups also increased significantly relative to those of their control lines, they were always lower by more than 4.0% than those of the corresponding CAU5-backcrossed new inducer lines (Figures 1, 3; Supplementary Tables 1, 2). One possible explanation for this result is that the CAU5 germplasm contains another major QTL related to the HI ability, *qhir8*, and it was retained due to our selection strategy, while the *qhir8* locus was congenitally absent in the CAUHOI genetic background, as well as the transformable lines used in this study (Prigge et al., 2012; Liu et al., 2015; Zhong et al., 2019).

Previous studies suggested that haploid formation in plants may be related to the centromere size of both parents in crosses (Britt and Kuppu, 2016; Wang and Dawe, 2018; Wang et al., 2019). According to the centromere size model, expression of an exogenous CENH3 tagged with a fluorescent protein in the native *cenh3* mutant lines may produce a defective CENH3 protein with lower loading capacity, thereby resulting in the genomic elimination of the inducer line due to its smaller centromere size when outcrossed to the wild-type line. Furthermore, modification of the N-terminal tail of this exogenous CENH3 protein will further decrease the strength of the assembled centromere, leading to an increase in the frequency of uniparental genome elimination (Kalitsis et al., 2003; Ravi et al., 2011; Wang and Dawe, 2018). Nevertheless, plants hardly induce haploids when coexpressing native and exogenous engineered *CENH3* genes *in vivo*, which may be related to the competing loading mechanisms of the CENH3 protein (Ravi and Chan, 2010; Karimi-Ashtiyani et al., 2015; Britt and Kuppu, 2016; Kelliher et al., 2016; Wang et al., 2019). Similarly, in this study, neither the original nor the tail-altered *CENH3-YFP/-RFP* overexpression lines could induce haploids when they were outcrossed to tester lines (Table 1), whether as male or female parents, which may be due to the retention of the native *CENH3* gene in these transgenic lines. In contrast, we found that introduction of modified *CENH3* overexpression vectors into Stock6-derived inducer lines could significantly enhance their maternal HI ability, especially by overexpressing the tail-altered CENH3 chimeric protein. Despite the *CENH3* coding sequence of the Stock6-derived lines was identical to that of the non-inducer lines (Zhao et al., 2013), a recent study revealed that spermatid chromosome fragmentation occurred in the developing pollen of Stock6-derived inducer lines, and this situation was mainly concentrated in the centromere regions (Li et al., 2017). This implies that the centromere function of the Stock6 germplasm may contain some undiscovered defects, possibly showing a similar consequence as a defective CENH3, thereby making it possible that overexpressing modified *CENH3* in the Stock6-derived lines could produce an additional HI ability and thus improve the HIR in the newly bred inducer lines (Marimuthu et al., 2021). Another possibility is that *CENH3* is involved in the underlying regulatory mechanism of chromosome fragmentation in Stock6 germplasms. Overexpression of an additional structural variant of the CENH3 protein in Stock6-derived inducer lines might aggravate chromosome fragmentation in developing pollen, thereby producing more defective pollen that has potential HI ability and resulting in an HIR increase in these engineered Stock6-derived lines.

It is worth to note that the native *CENH3* gene is still retained and functional in our newly bred inducer lines, which may have a negative impact on the improvement of HIR based on the assumptions above. Given recent progress, homozygous *cenh3* mutations in maize are generally lethal to plants (Feng et al., 2020; Wang et al., 2021). Therefore, knocking down the expression level of native *CENH3* in these engineered Stock6-derived inducer lines may be an effective way to further improve their maternal HIRs. Moreover, our results showed that there was no significant difference in the increase in HIR between the constructs with the YFP and RFP tags, which may be due to the relatively similar sizes of the two protein tags used in this study (239 amino acids of YFP and 237 amino acids of RFP). Thus, we can try to use more types of tags with different sizes to fuse the CENH3 protein in the next step and determine the optimal tag for HIR improvement.

In summary, our results showed that two *in vivo* HI approaches mediated by Stock6 germplasm and genetically engineered *CENH3* could be well integrated to promote maternal HIRs improvement in maize haploid inducer lines, which offers a potential solution for further optimizing the maize DH breeding process. Moreover, with the development of HI-related research, this combinatorial method may be further improved to make it more efficient and simpler. For example, with more Stock6 germplasm-related HI genes have been cloned, we can simultaneously manipulate *CENH3* and multiple HI genes in a same genetic transformation receptor, thereby creating high-HIR inducer lines with homozygous genetic backgrounds in a shorter period.

## Materials and Methods

### Vector Construction and Genetic Transformation

Four plasmid constructs were prepared in this study: *p35S::CENH3-YFP*, *p35S::C-tailswap-YFP*, *pUBI::CENH3-RFP*, and *pUBI::M-tailswap-RFP*. For the *p35S::CENH3-YFP* and *pUBI::CENH3-RFP*, the full-length coding sequence of the maize *CENH3* gene (*Zm00001d038533*; Jiao et al., 2017) was amplified from the cDNA of LH244 leaves and cloned into the HindIII and BamHI sites of the overexpression vector *pCM3301M-YFP* driven by the *CaMV 35S* promoter and the XbaI and KpnI sites of the overexpression vector *pCM3300M-RFP* driven by the *UBIQUITIN (UBI)* promoter, respectively. For the *p35S::C-tailswap-YFP* vector, the maize CENH3 N-terminal tail sequence (1–60 amino acids) of coix was replaced with the N-terminal tail sequence (1–61 amino acids) of coix CENH3 (Cl029833; vCAU v1.0; Liu et al., 2020), the fragments of 183 bp 5′ end of coding sequence of coix *CENH3* and 291 bp 3′ end of coding sequence of maize *CENH3* were PCR amplified from the cDNA of coix and maize LH244 leaves, respectively. The PCR products were cloned into the *pCM3301M-YFP* construct by using the HindIII, AscI, and BamHI sites to generate the *p35S::C-tailswap-YFP* vector driven by the *CaMV 35S* promoter. For the *pUBI::M-tailswap-RFP* vector, the maize CENH3 N-terminal tail sequence (1–60 amino acids) was replaced with the N-terminal tail sequence (1–42 amino acids) of maize H3.2 (Zm00001d050697; Jiao et al., 2017), the fragments of 126 bp 5′ end of coding sequence of maize *H3.2* and 291 bp 3′ end of coding sequence of maize *CENH3* were PCR amplified from the cDNA of maize LH244 leaves. The PCR products were cloned into the *pCM3300M-RFP* construct by using the XbaI, SpeI, and KpnI sites to generate the *pUBI::M-tailswap-RFP* vector driven by the *UBIQUITIN* promoter. In addition, the empty vector *pCM3301M-YFP* (referred to as *p35S::YFP*) was used as a control.

All the constructs were introduced into the maize transformable line LH244 or Hill using the *Agrobacterium-*mediated method (Ishida et al., 2007). *T*_0_ generation plants were screened using specific molecular markers *via* PCR technology, and the positive transgenic plants were self-pollinated to generate *T*_1_ homozygous progenies (referred to as LH244^35S::YFP^, LH244^CENH3-YFP^, LH244^CENH3-RFP^, LH244^M-tailswap-RFP^, and HiII^C-tailswap-YFP^).

### Cross Strategy

The *T*_1_-positive transgenic plants obtained from previous genetic transformation were backcrossed with CAU5 and CAUHOI for 3 generations and then self-pollinated for 4 and 2 generations, respectively. In each generation, using the specific molecular markers qhir1-*P*1 and bar-*P*1, we randomly screened 25 plants containing both the *qhir1* locus and CENH3 fusion protein from each backcross combination to test the maternal HIR: each selected plant was self-pollinated and simultaneously used to pollinate three tester ears for determination of its HIR. Then, the self-pollinated seeds of three plants with the highest HIRs among the 25 plants were selected for backcrossing or selfing in the next generation. The selection strategy for the non-transgenic control lines (CAU5^LH244-introgressed^, CAUHOI^LH244-introgressed^, CAU5^HiII-introgressed^, and CAUHOI^HiII-introgressed^) was the same as described above, except that only one locus of *qhir1* was screened during each selection cycle.

All plant materials used for backcrossing, selfing, and haploid identification were grown in the field at the Experimental Station in Shangzhuang, China Agricultural University, Beijing.

### Haploid Identification and HIR Calculation

Haploid identification was carried out according to a previously described method (Li et al., 2009; Dong et al., 2013). The hybrid Zhengdan958 was chosen as the tester for evaluating the maternal HIRs of all the inducer lines used in this study. Since both the donor inducers CAU5 and CAUHOI carried the *R1-nj* gene as the pigmentation marker, mature kernels would be classified as diploids if their embryo and endosperm showed purple coloration in the testcross progenies with the tester Zhengdan958, and kernels with colorless embryo and purple endosperm would be classified as putative haploids. All putative haploids were planted in the field in the next planting season to confirm their ploidy status by evaluating their agronomic traits, such as the plant height, the length, width and angle of leaves, and pollen fertility.

The HIR was calculated using the following formula: HIR = (number of haploids in the field/total number of *R1-nj* normal kernels) × 100% (Dong et al., 2013). The population HIR of each selection generation of each newly bred inducer line was calculated through statistical analysis of individual HIR of the 25 selected plants.

### qRT-PCR Assay

The leaves of the *s*_1_ homozygous plants and transformable receptor plants were sampled, and total RNA was extracted using a Quick-RNA isolation kit (Huayueyang Biotechnology). cDNA was synthesized with a reverse transcription (RT) reagent kit (Invitrogen) according to the manufacturer’s instructions. qRT-PCR was conducted on a CFX96 real-time system (Bio-Rad) with a TB Green RT-PCR kit (Takara). Relative gene expression levels were calculated according to the 2^–ΔΔCt^ relative quantification method with maize *ACTIN (Zm00001d010159)* as the internal control (Livak and Schmittgen, 2001).

### TTC Staining

Three inducer lines, namely, CAU5, CAU5^C-tailswap-YFP^, and CAU5^M-tailswap-RFP^, were chosen to measure pollen viability using the TTC staining method. The fresh pollen of the above inducer lines was separately collected from the field at 9:00 a.m. on the same day, and then, the samples were incubated in a 1.5 ml tube with 0.1% TTC at 37°C for 0.5–1 h. The viability of the stained pollen was determined, and the pollen was photographed using a Carl Zeiss Axio Zoom V16 stereomicroscope. The classification of pollen viability was carried out according to previously described methods (Li et al., 2017). The pollen grains were collected three times on different days as biological replicates for pollen viability detection. More than 30,000 pollen grains were counted for each tested inducer line.

### Primers

All primers used in this study were listed in Supplementary Table 3.

## Data Availability Statement

The original contributions presented in the study are included in the article/Supplementary Material, further inquiries can be directed to the corresponding authors.

## Author Contributions

WJ and HY conceived and designed the experiments. DM and HL performed most of the experiments, data analysis, and wrote the article. SC provided the CAU5 and CAUHOI seeds. FaL and FeL provided technical assistance. WJ, HY, ZD, and WH edited the manuscript. All authors contributed to the article and approved the submitted version.

## Funding

This research was supported by the National Natural Science Foundation of China (31801368 and 92035302) and China Postdoctoral Science Foundation (2021M692236).

## Conflict of Interest

The authors declare that the research was conducted in the absence of any commercial or financial relationships that could be construed as a potential conflict of interest.

## Publisher’s Note

All claims expressed in this article are solely those of the authors and do not necessarily represent those of their affiliated organizations, or those of the publisher, the editors and the reviewers. Any product that may be evaluated in this article, or claim that may be made by its manufacturer, is not guaranteed or endorsed by the publisher.
