# Supplementary material for: Overexpression of Modified CENH3 in Maize Stock6-Derived Inducer Lines Can Effectively Improve Maternal Haploid Induction Rates
Source: Front Plant Sci. 2022 Apr 11;13:892055. doi: 10.3389/fpls.2022.892055 (PMC9036175; doi:10.3389/fpls.2022.892055)
Supplement: Supplementary file 4 [file Image_1.PDF]

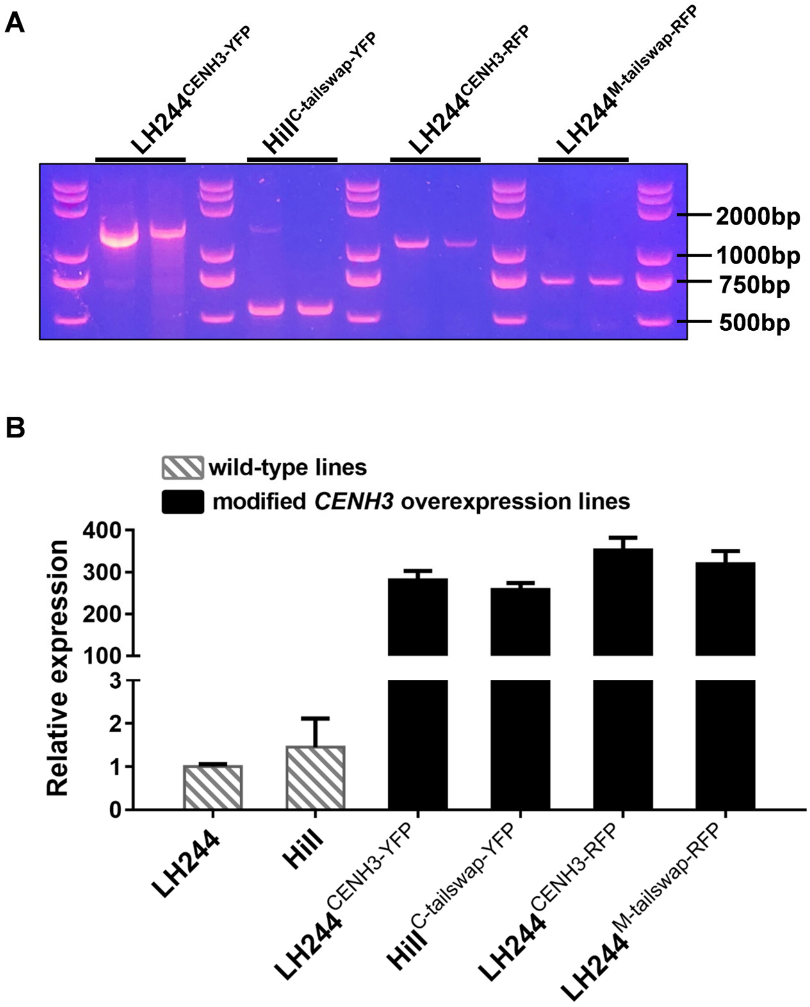


**Supplementary Figure 1. Identification of positive transgenic plants using PCR and qRT-PCR methods.**

(A) Identification of positive transgenic plants by PCR technology using specific primers. The DNA bands indicate the transformation vector fragments amplified by specific primers.

(B) Expression level of *CENH3* (*Zm00001d038533*) in the wild-type and overexpression lines.

Error bars indicate ±SD.
