# Supplementary material for: Overexpression of Modified CENH3 in Maize Stock6-Derived Inducer Lines Can Effectively Improve Maternal Haploid Induction Rates
Source: Front Plant Sci. 2022 Apr 11;13:892055. doi: 10.3389/fpls.2022.892055 (PMC9036175; doi:10.3389/fpls.2022.892055)
Supplement: Supplementary file 5 [file Image_2.PDF]

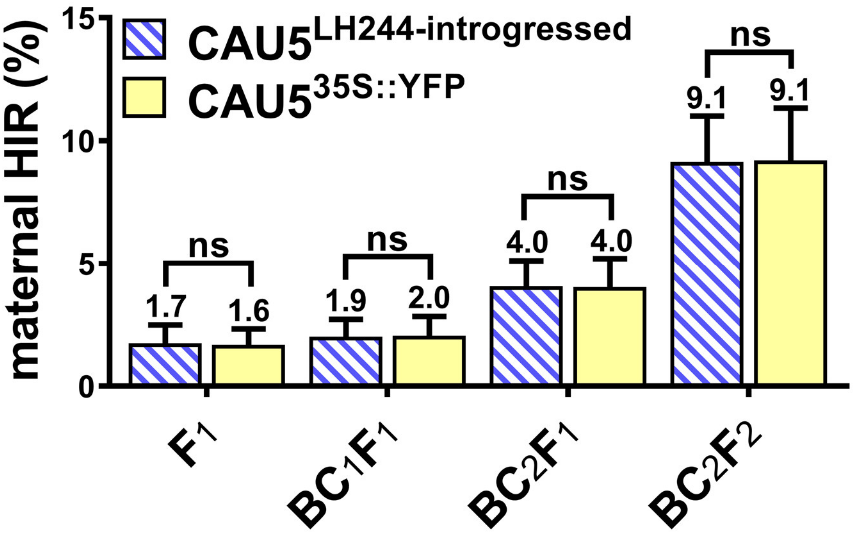

Supplementary Figure 2. Comparison of maternal HIR between the CAU5$^{LH244\text{-}introgressed}$ and CAU5$^{35S::YFP}$ inducer lines in consecutive breeding generation.

The number above the column indicates the average maternal HIR. Error bars indicate ±SD. Significant differences were analyzed by two-tailed Student's t tests (ns, not significant).
